# Mechanical strength and shape accuracy optimization of polyamide FFF parts using grey relational analysis

**DOI:** 10.1038/s41598-022-17302-z

**Published:** 2022-07-30

**Authors:** Zohreh Shakeri, Khaled Benfriha, Nader Zirak, Mohammadali Shirinbayan

**Affiliations:** 1 Laboratoire Conception de Produits et Innovation (LCPI), HESAM University, 75013 Paris, France; 2 Procédés Et Ingénierie en Mécanique et Matériaux (PIMM), HESAM University, 75013 Paris, France

**Keywords:** Engineering, Materials science, Mathematics and computing

## Abstract

This paper investigates the effect of different additive manufacturing process parameters such as chamber temperature, Printing temperature, layer thickness, and print speed on five essential parameters that characterize the manufactured components: cylindricity, circularity, strength, and Young’s modulus, and deformation by Gray Relational Analysis method simultaneously. Taguchi method was used to design the experiments, and the PA6 cylindrical parts were fabricated using a German RepRap X500® 3D printer. Then the Gray Relational Grade (GRG) values were calculated for all experiments. In the 8th trial, the highest value of GRG was observed. Then, to discover the optimal parameters, the GRG data were analyzed using ANOVA and S/N analysis, and it was determined that the best conditions for enhancing GRG are 60 °C in the chamber temperature, 270 °C in the printing temperature, 0.1 mm layer thickness, and 600 mm/min print speed. Finally, by using optimal parameters, a verification test was performed, and new components were investigated. Finally, comparing the initial GRG with the GRG of the experiments showed an improvement in the gray relational grade (14%) which is accompanying with improving of GRG value.

## Introduction

One of the most widely used additive manufacturing (AM) methods is Fused Filament Fabrication (FFF), which can produce complex geometry parts. In this process, a continuous filament of thermoplastic material is deposited layer by layer to make the final piece. One of the essential goals in the production of parts with this method is to produce parts with high mechanical properties and excellent geometrical accuracy at the same time. Among the various methods used to improve the desired properties of produced parts is to determine the optimum process parameters in this process^1–3^.

ME, also known as Fused Filament Fabrication (FFF) as described in the ISO/ASTM 52900, is a popular AM method that involves selective deposition due to less material wastage, less expensive materials, and tools of thermoplastic polymers through a heated nozzle. As a 3D printing technique in prototype and end-product applications among additive manufacturing methods^4,5^.

In the FFF process, a polymer is supplied into a liquefier, which extrudes a filament while moving in successive X–Y planes along the Z-axis to layer-by-layer build a 3D object^6,7^. Polylactide (PLA), Polyamide (PA), Polycarbonate (PC), Acrylonitrile Styrene Acrylate (ASA), Nylon, Acrylonitrile Butadiene Styrene (ABS) and, Polyether Ether Ketone (PEEK) seem to be the most often utilized thermoplastic polymers used in the FFF technique to make 3D parts^8^. The most significant disadvantages of this approach include poor surface quality, slow build speed, and anisotropic mechanical properties resulting from the layer-by-layer strategy^9,10^. Due to 3D printed parts usage, mechanical behavior and geometrical accuracy must be carefully examined to avoid wasting materials and time^11^.

Because many parameters such as might impact the printing process, manufacturers' default configuration of printing process parameters cannot guarantee the quality of printed products^12^. Various process parameters such as nozzle temperature, layer thickness, shell thickness, printing speed, infill density and other parameters with multiple responses, control the FFF process and they should be optimized to improve the required quality. From the perspective of analysis, this makes it a rather tricky procedure. Therefore, extensive research is being conducted to determine the impact of various FFF process parameters on the different responses^13^.

Ju-Long^14^ developed the Gray Relational Analysis (GRA), which is one of the multi-response optimization techniques, and it is based on the Taguchi technique. Many recent studies based on the Gray Relational Analysis (GRA) have been performed to improve different responses through processing parameter optimization. For example, Venkatasubbareddy et al.^15^, used the Taguchi method with Grey Relational Analysis (GRA) to determine the best combination of FDM process characteristics for ABS parts, resulting in improved surface finish and dimensional accuracy in terms of length, thickness, and diameter. L27 Orthogonal Array was chosen for this experiment using Taguchi’s DOE with five parameters: air gaps, layer thickness, raster width, raster angle, And part orientation at three levels of each parameter. It was stated that the layer thickness of 0.254 mm, part orientation and raster angle of 0°, raster width of 0.4564 mm, and zero air gaps should improve the components’ surface quality and dimensional accuracy. Aslani et al.^16^ investigated the impact of the number of shells, printing temperature, infill rate, and printing pattern on the dimensional accuracy of the PLA. The Grey–Taguchi technique with ANOM and ANOVA techniques was used to determine the optimum printing parameter levels for PLA FFF components, resulting in the best dimensional accuracy. Regarding dimensional deviations in two dimensions, a multi-response optimization was carried out and obtained results showed that the essential characteristic, according to the data, is the nozzle temperature. Furthermore, Analysis shows that the levels that minimize dimensional deviation are three shells, 230 °C Printing temperature, one of the recommended printing temperatures of PLA, 10 % Infill rate, and hexagonal printing pattern.

Deng et al.^17^, analyzed the effect of 4 parameters, including printing speed (20, 40 and 60 mm/s), layer thickness (0.2, 0.25 and 0.3 mm), printing temperature (350, 360 and 370 °C), and filling ratio (20, 40 and 60 %) on strain, strength, and stiffness. The substance studied in this study is polyether-ether-ketone (PEEK) which is manufactured by FFF. It was observed that the mechanical properties would increase in printing speed of 60 mm/s, the layer thickness of 0.2 mm, the temperature of 370 °C, and the filling ratio of 40%. Also, they observed the microstructure of PEEK parts. They understood that there is more air gap and fusion lines in the part with lower strength, so it was concluded that lower printing temperature and printing speed caused more defects in the part. In addition, the connectivity could be influenced by a subcooled temperature between the fused filament, chamber, and bed temperatures. Xiaoyong et al.^18^ investigated the effect of bed temperature (130, 110 and, 25 °C), chamber temperature (60 and 25 °C) and filling ratio (50% and 100%) on mechanical properties and forming precision, the sheet forming of PEEK thermoplastic parts fabricated using FFF method. They understood that temperature significantly impacts mechanical properties, and increasing the temperature can improve mechanical qualities. In the higher bed and chamber temperature, the tensile strength and printing precision will be enhanced due to the increase in bonding force between the layers. Also, mechanical properties are improved at low filling ratios. By comparing the PEEK and PLA strength results, it was discovered that the tensile strength of PEEK parts is higher than previously thought. Aamir et al.^19^ applied the Taguchi and GRA technique to determine the effects of five parameters: raster width, layer thickness, printing speed, and extrusion temperature on build time, surface roughness, and flatness error of PC/ABS blend parts. L27 Orthogonal Array of Taguchi’s design of experiments Selected and GRA techniques were used to select the optimum FDM variables for responses using multi-objective optimization. According to the investigation results, Raster width, layer thickness, and printing speed significantly impact multiple control factors. The layer thickness of 0.2 mm, the Raster width of 0.55 mm, extrusion temperature 270 °C, Bed temperature 100 °C, and Printing speed 40 mm/s are optimal conditions. Using the full factorial design methodology, Kechagias et al.^20^ investigated the effect of Raster deposition angle, cutting speed, Laser power, and Stand-off distance on dimensional accuracy and surface roughness using a low-power CO2 laser cutting of thin acrylonitrile butadiene styrene (ABS) plates manufactured with the FFF process. Finally, ANOVA analysis, interaction studies, and quadratic regression models were used to match input and output parameters, optimize the process kerf angle near 0°, and decrease surface roughness near 0 m. The best prosses parameters were 7.5 mm stand-off distance, zero raster deposition angle, 14.4 mm/s laser speed, and 105 W laser power. Anusree et al.^21^ analyzed the effects of four variables, including print speed, layer thickness, support material density, and raster width, on dimensional accuracy, tensile strength, and surface finish of FDM-processed helical surfaces using Taguchi and GRA methods. It was stated that the better dimensional accuracy, tensile strength, and surface finish were obtained by a minimum level of the layer thickness, in a print speed of 58 mm/s, and maximum level of raster width and rough support material. In N. Vidakis et al.^22^ research examined the influence of layer height (0.15, 0.2, and 0.25 mm), nozzle temperature (250, 260, and 270 °C), and raster angle (0, 45, and 90°) on the mechanical strength and toughness of FFF Polyamide 12 polymer using a full factorial methodology. Experimental results were analyzed using ANOVA analysis, interactive diagrams, and box diagrams, and it was determined that experimental results were analyzed using ANOVA analysis, interaction, and box diagrams. It was found that while 270°C optimizes static mechanical strength and elastic modulus and at 260 °C, the toughness and zero degrees raster angle, static mechanical strength, and toughness values ​​are optimized at 45 degrees raster angle elastic modulus values ​​are optimized. In addition, all responses are optimized with a layer height of 0.25 mm. Kechagias et al.^23^ used the Taguchi L18 orthogonal array to analyze the effect of the nozzle temperature (180, 200, and 220 °C), layer thickness (0.1 and 0.3 mm), filament printing speed (30, 40, and 50 mm/s), and deposition angle (0, 45, and 90°) on mechanical properties such as ultimate tensile strength (UTS) and elasticity module (E) of PLA/Coconut wood parts. The results were analyzed using ANOM and ANOVA methods. The raster deposition angle has the most significant effect on responses, and the zero-oriented filament has greater UTS and E values (80.1% on UTS and 92.6% on E). The layer thickness is a significant parameter; although it is highly dependent on the interlaminar bonding state, the effect of this parameter was insignificant in this research. Also, they understood the mechanical behavior in various layer thicknesses is different by changing the printing speed. In our previous research^24^, the effect of four parameters, including thickness (5, 10, and 15 mm), infill pattern (Hexagonal, Rectangular, Triangular), number of walls (2, 3, and 4), and Layer height (1, 1.125 and 2 mm) were analyzed on geometrical accuracy of cylindrical PA6 parts using Taguchi method. It was understood the effect of thickness and Layer height is more significant. The best process parameters for minimum geometrical error were the hexagonal infill pattern, a thickness of 5 mm, a wall layer of 2, and a layer height of 1.125 mm. According to N. Vidakis et al.^25^ nozzle temperature and layer height values affect ultimate tensile strength, elastic modulus, average, and the max-min differences ultimate tensile strength and elastic modulus (Δ*σb* and Δ*E)* were observed using the general full factorial method. After it was revealed that in the nozzle temperature of 270 °C and a layer height of 0.2 mm, all the responses were optimized (maximizes the ultimate tensile strength and elastic modulus and minimizes the Δ*σb* and Δ*E).*

In the studies mentioned in the literature, the effects of different parameters in FFF on mechanical properties and shape accuracy on different materials were investigated. However, no research in the literature was not reported where the influence of four parameters, such as the chamber temperature, Printing temperature, layer thickness, and print speed, on the mechanical strength and shape accuracy of the PA6 simultaneously. Therefore, this work is an experimental investigation of how these four FFF parameters, including Chamber temperature (30, 45, and 60 °C), Printing temperature (260, 270, and 280 °C), layer thickness (0.1, 0.2, and 0.3 mm), and print speed (600, 1800 and 3000 mm/min), on the mechanical properties and geometrical accuracy of cylindrical parts made of PA6, produced by the FFF process. The results were analyzed using the GRA method and presented using S/N analysis, ANOVA analysis, interaction plots, counter and surface plots, and a regression model. Finally, a confirmation test was performed to validate the results. In the final part of this work, the results obtained were discussed.

## Materials and methods

### Experimental setup

**Figure 1:**
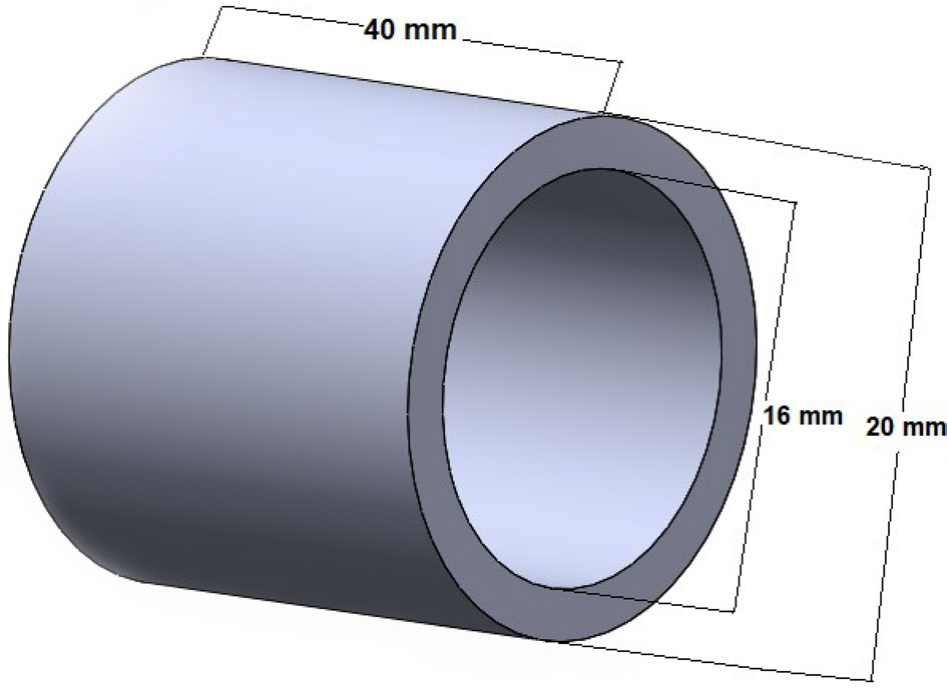
Hollow cylindrical dimensions.

**Figure 2:**
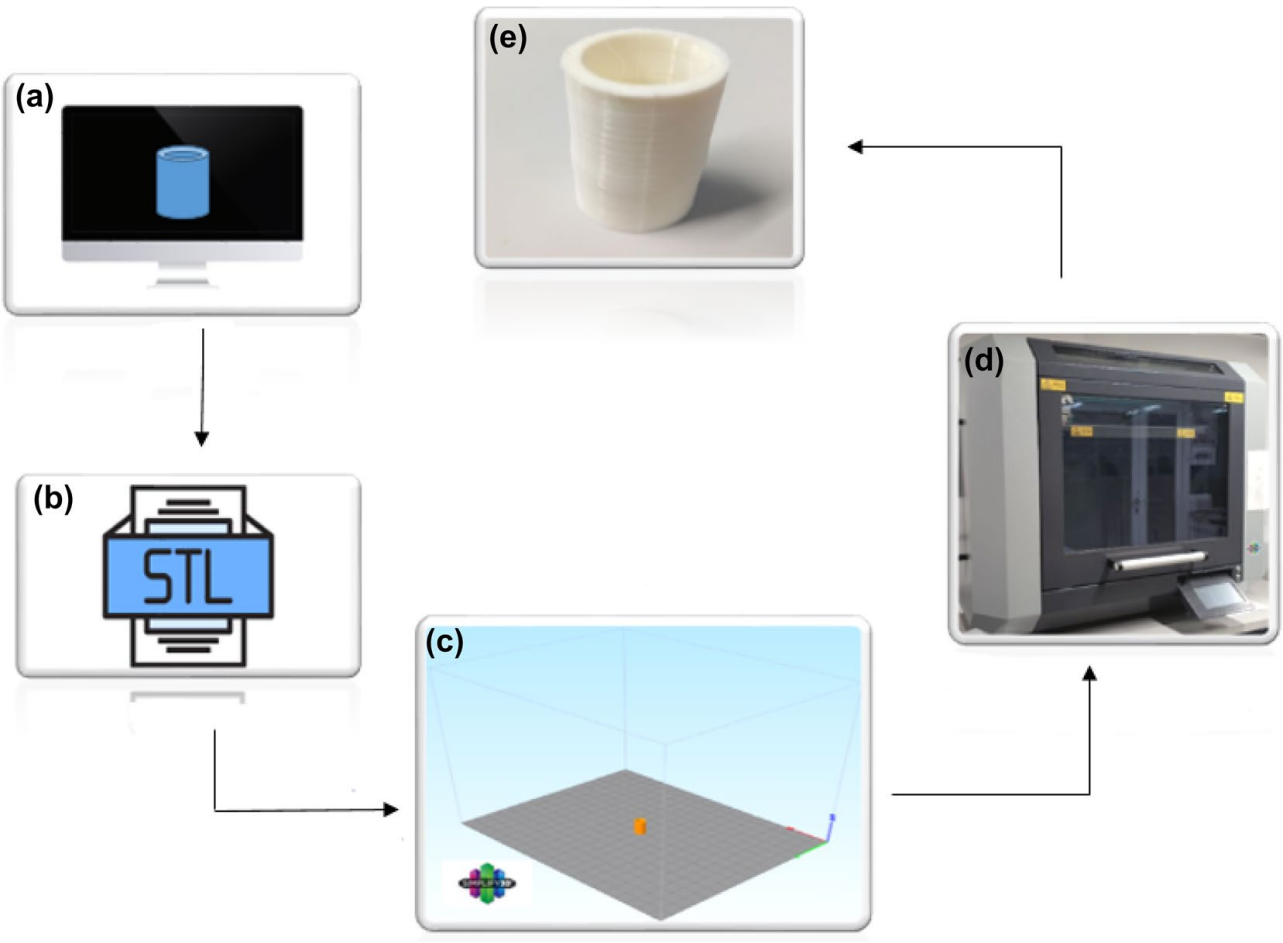
Schematic of 3D printing steps. (**a**) Creating a digital model, (**b**) converting the digital model to STL, (**c**) slicing and choosing the presses parameters, (**d**) printing the parts by German Rep Rap 3d printer, (**e**) 3d printed cylindrical parts.

In this study, the PA6 samples were fabricated by a German RepRap X500^®^ 3D printer, which uses fused filament fabrication (FFF) technology. This machine has a high degree of design freedom and allows designers to experiment with completely new design and functionality concepts. Some of the technical specifications of the German RepRap X500^®^ 3D printer are shown in Table 1. Nylon white or PA6 is one of the most widely used polyamides. Also, it is a commercial material with high surface quality and excellent mechanical properties, so we chose this material in this research^26–28^. A hollow cylindrical part with the dimensions of inner diameter 16 mm and outer diameter 20 mm with a height of 40 mm was designed (Fig. 1) using CATIA-V5™ software and exported as an STL file. After slicing the parts with Simplify 3D software used to set FFF parameters, they will be manufactured using a German RepRap X500^®^ 3D printer. Figure 2 shows a schematic of these steps.

**Table 1:** Technical specifications for German RepRap X500®printer.

	Specifications
Extruder	Dual extruder with dual lift extruder system
Software setup	Simplify3D slicer software
Build volume	500 × 400 × 450 mm
Print speed	600–9000 mm/min
Extruder temperature (Max)	400 °C
Chamber temperature	80 °C

### Design of experiment (DOE)

Design of Experiments (DOE) is a systematic approach for determining the effect of input process parameters on a single or set of output responses in the setting of process parameter optimization. Several DOE approaches have been utilized to optimize the process parameters of the FFF system, including the Taguchi method, analysis of variance (ANOVA), complete factorial designs, bacterial foraging technique, which was recently created and has received a lot of attention due to its efficiency in solving real-world optimization problems, is a nature-inspired optimization algorithm based on Escherichia coli bacteria foraging behavior, and fuzzy logic because many parameters might impact the printing process, the default configuration of printing process parameters provided by manufacturers cannot guarantee the quality of printed products^29,30^. Extensive research is being conducted to determine the impact of various FFF process parameters on the different responses^31–34^. The Taguchi design method provides a practical approach to lower cost, higher quality, and performance optimization. In the Taguchi design technique, many parameters may be analyzed at once, and the best optimal configuration can be found with fewer resources than in the traditional DOE approach.

The L9 Orthogonal Array used in this study and the effect of four critical parameters of the FFF process, including layer thickness (mm), print speed (mm/min), chamber temperature (°C), and print temperature (°C) in three different levels investigated on cylindricity and circularity as geometric accuracy. Also, Young’s modulus, strength, and deformation as mechanical properties were analyzed on these samples.

Process parameters in three different levels are shown in Table 2. The chamber controlled the environment temperature, and the chamber temperature was set at 30, 40, and 60 °C. Layer thickness refers to the thickness of each deposited layer and is based on the dimensions of the cylinders, and it was selected in the range of 0.1, 0.2, and 0.3 mm. The print speed was set low to high at 600, 1800, and 3000 mm/min. Because the print temperature of nylon is usually 270 °C^35^, the selected temperature was slightly higher and lower than 270 °C to investigate the responses (250, 260 and, 270 °C). Table 3 shows the Taguchi orthogonal array that controls the parameter combinations for each experiment. Also, to increase the repeatability, each part has been printed five times, and 45 pieces have been fabricated.

**Table 2:** The process parameters and their levels.

Level	Chamber temperature (°C)	Printing temperature (°C)	Layer thickness (mm)	Print speed (mm/min)
1	30	260	0.1	600
2	45	270	0.2	1800
3	60	280	0.3	3000

**Table 3:** L9 orthogonal array.

No. of trial	Chamber temperature (°C)	Printing temperature (°C)	Layer thickness (mm)	Print speed (mm/min)
1	30	260	0.1	600
2	30	270	0.2	1800
3	30	280	0.3	3000
4	45	260	0.2	3000
5	45	270	0.3	600
6	45	280	0.1	1800
7	60	260	0.3	1800
8	60	270	0.1	3000
9	60	280	0.2	600

### Measurement of responses

**Figure 3:**
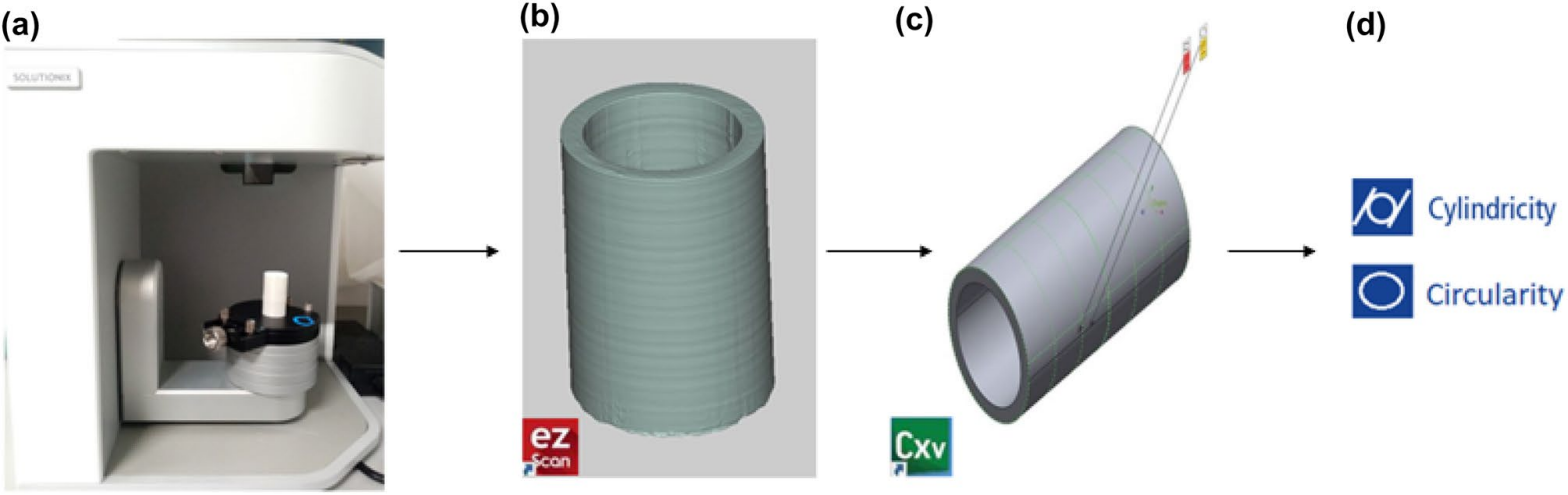
Schematic of scanning process to find the cylindricity and circularity error. (**a**) Scaning the parts by Solutionx D500, (**b**) Solutionx Ezscan software, (**c**) Geomagic Control X, (**d**) Measuring the geometrical accuracy.

First, all 3D printed parts were scanned using a 3D laser scanner (Solutionix D500) to measure geometrical error values. With an accuracy of 0.01 mm and a resolution of 0.055 mm. The advantage of this scanner is the high speed of scanning processing. The process consisted of using the blue light reflected from the object’s surface to the camera lens from a blue light source projected onto the surface of the parts. Then it is reflected from the object’s surface to the camera lens. Point-by-point coordinates are displayed in Solutionix ezScan software, which controls Solutionix D500, and the geometry of the part Obtained. This data was then extracted in STL format from Solutionix ezScan. In the next step, the initial CAD model was compared with the STL files extracted from Solutionix ezScan by Geomagic^®^ Control X software and aligned by component alignment. Finally, the circularity and cylindricity errors were obtained based on the ASME Y14.5M standard. The measured values of cylindricity and circularity are shown in Table 4, and the schematic of the steps is shown in Fig. 3.

**Table 4:** Measured values of responses.

No. of trial	Young’s modulus (MPa)	Strength (MPa)	Deformation	Cylindricity (mm)	Circularity (mm)
1	1564.98 ± 50.86	62.50 ± 0.39	0.0556 ± 0.00045	0.177 ± 0.01108	0.136 ± 0.0118
2	1327.24 ± 36.49	52.12 ± 0.47	0.1164 ± 0.000741	0.258 ± 0.00242	0.194 ± 0.01439
3	1346.30 ± 12.27	51.21 ± 0.217	0.0883 ± 0.00171	0.219 ± 0.01605	0.154 ± 0.01315
4	1401.54 ± 6.572	51.42 ± 0.225	0.0635 ± 0.000497	0.206 ± 0.00695	0.151 ± 0.0060
5	1594.29 ± 25.106	59.48 ± 0.370	0.1256 ± 0.000064	0.243 ± 0.01715	0.245 ± 0.01187
6	1592.60 ± 3.878	64.48 ± 0.253	0.056 ± 0.000427	0.236 ± 0.02378	0.203 ± 0.02284
7	1313.97 ± 6.673	47.73 ± 0.202	0.062 ± 0.000682	0.261 ± 0.008515	0.169 ± 0.00090
8	1685.25 ± 7.168	63.36 ± 0.349	0.0539 ± 0.00022	0.180 ± 0.01212	0.146 ± 0.0046
9	1730.69 ± 5.575	65.54 ± 0.825	0.0595 ± 0.000679	0.243 ± 0.0174	0.174 ± 0.01277

**Figure 4:**
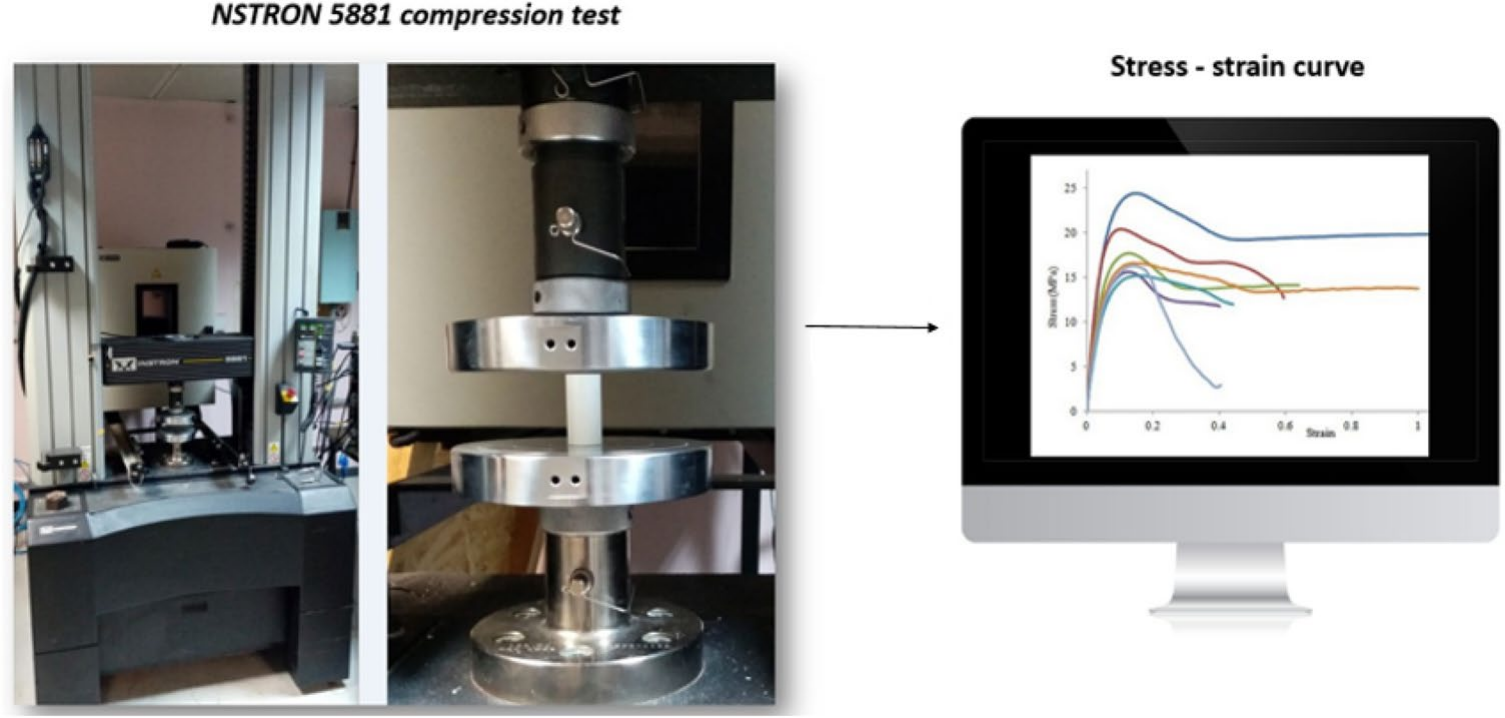
Steps to find the strength, Young’s modulus, and elongation.

**Figure 5:**
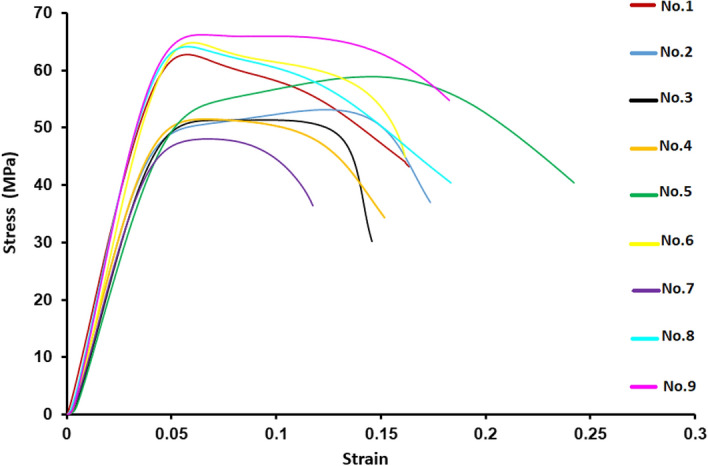
Stress–strain curves of all trials.

**Figure 6:**
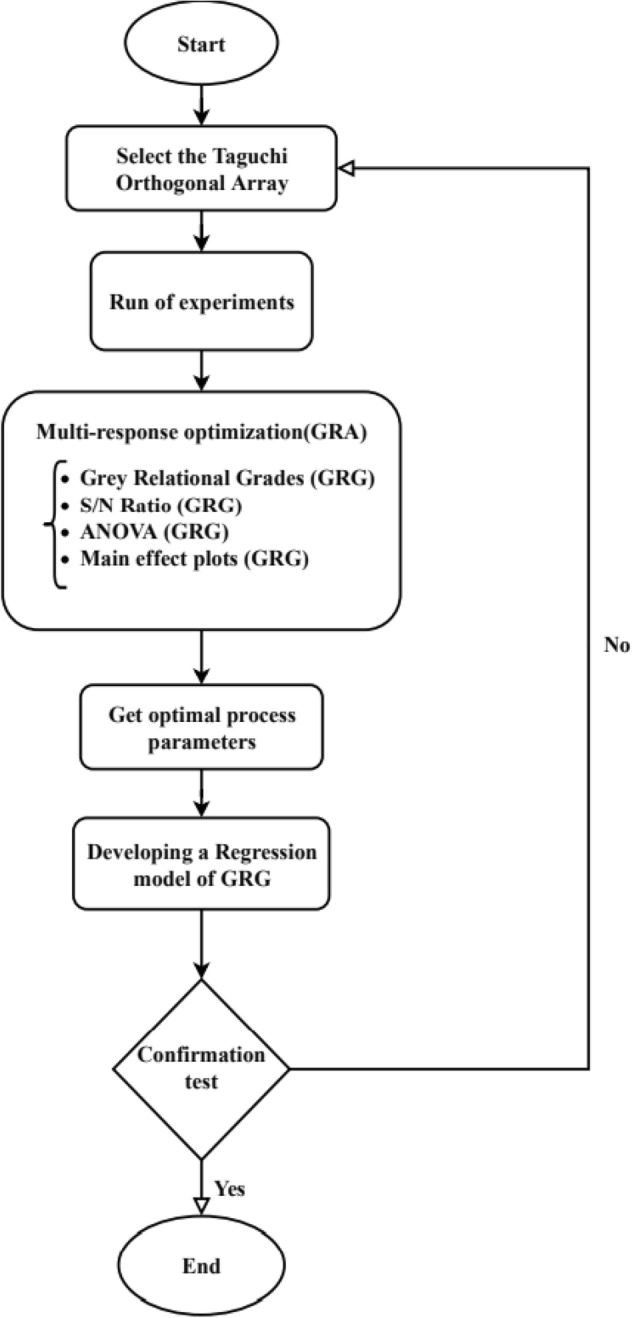
Flowchart of implementing the steps.

The mechanical properties of parts were measured by compression test (INSTRON 5881 compressive and tensile testing machine), all the samples were compressed using a loading cell of 50 KN and loading speed of 5 mm/min. The special jaws were designed to perform the compression tests, and the tubes were positioned between two jaws as sketched, as shown in Fig. 4. Then a stress-strain curve was obtained (Fig. 5). Strength, Young’s modulus, and elongation are shown in Table 4, and the steps are shown in Fig. 5 a flowchart shows the steps of the optimization process (Fig. 6).

## Results and discussion

Experiments' data were investigated one by one by analysis of variance (ANOVA) and Signal-to-noise ratio (S/N). MINITAB^®^ 19.0 was used to analyze all of the data. Taguchi method is used to investigate the effect of a large number of parameters on a certain response with a fewer number of experiments.

### Multi-response optimization

The GRA method was simultaneously used to optimize multi-response parameters, a statistical method. This method simultaneously reduces the cylindricity and circularity and increases the strength, elongation, and young’s modulus by calculating the optimal process parameters. GRA is applied in the following steps.

#### Normalization of experimental data

The first step is to normalize the experimental data. According to the expected quality characteristics of different responses, this value can be divided into three criteria for optimization in GRA: “larger-is-better,” “smaller-is-better,” and “normal-is-better” are shown in Eqs. (1, 2 and 3) ^36^.

Larger-is-better:

1 $${X}^{*}(p)=\frac{{X}_{i}(p)-Min{(X}_{i}(p))}{Max{(X}_{i}(p))-Min{(X}_{i}(p))}$$

Smaller-is-better:

2 $${X}^{*}(p)=\frac{Min{(X}_{i}(p)){-X}_{i}(p)}{Max{(X}_{i}(p))-Min{(X}_{i}(p))}$$

Normal-is-better:

3 $${X}^{*}(p)=1-\frac{|{X}_{i}(p)-OB|}{Max{[Max(X}_{i}(p))-OB,OB-Min{(X}_{i}(p))]}$$

where X^*^ (p*)* is the GRG value, *i* shows the number of trials, *X*_i_*(*p*)* represents the response value of the target experiment, *Max(X*_i_*(*p*))* is the maximum value of *X*_i_*(*p*), Min(X*_i_*(*p*))* demonstrates the minimum value of *X*_i_*(*p*)* and *OB* is the target value. In this study, the “smaller-is-better” is chosen to normalize the cylindricity and circularity, and “larger-is-better” is chosen for strength, Young’s modulus, and deformation the normalized values are shown in Table 5.

**Table 5:** Normalized values.

No. of trial	Young’s modulus (MPa)	Strength (MPa)	Deformation	Cylindricity (mm)	Circularity (mm)
1	0.602	0.829	0.024	1.000	1.000
2	0.032	0.246	0.872	0.042	0.472
3	0.078	0.195	0.480	0.502	0.834
4	0.210	0.207	0.134	0.658	0.868
5	0.673	0.660	1.000	0.213	0.000
6	0.669	0.940	0.029	0.302	0.383
7	0.000	0.000	0.113	0.000	0.695
8	0.891	0.878	0.000	0.956	0.907
9	1.000	1.000	0.078	0.222	0.655

#### Deviation sequence

The next step is to calculate the deviation sequence from Eq. (4).

4 $${\Delta }_{oi} \left(p\right)=|| {X}_{0}\left(p\right)-{X}_{i}\left(p\right) ||$$

where *∆*_*oi*_* (p)* represents the deviation sequence and *X*_*0*_*(p)* is the reference sequence which is equal to one. Values of deviation sequence for each response are given in Table 6^36^.

**Table 6:** Deviation sequence of GRA.

No. of trial	Young’s modulus (MPa)	Strength (MPa)	Deformation	Cylindricity (mm)	Circularity (mm)
1	0.398	0.171	0.976	0.000	0.000
2	0.968	0.754	0.128	0.958	0.528
3	0.922	0.805	0.520	0.498	0.166
4	0.790	0.793	0.866	0.342	0.132
5	0.327	0.340	0.000	0.787	1.000
6	0.331	0.060	0.971	0.698	0.617
7	1.000	1.000	0.887	1.000	0.305
8	0.109	0.122	1.000	0.044	0.093
9	0.000	0.000	0.922	0.778	0.345

#### Grey relational coefficients

The relationship between ideal and real normal experimental results is expressed by the Gray Relational Coefficient (GRC). The Grey relationship coefficient is calculated using Eq. (5)^36^ for each of the normalized values.

5 $${X}^{*}\left(p\right)=1-\frac{{\Delta }_{min}+{\zeta .\Delta }_{max}}{{\Delta }_{oi}\left(p\right)+{\zeta .\Delta }_{max}}$$

where ζ_i_ (p) is Gray Relation Coefficient, *∆*_*oi*_* (p)* represents the deviation sequence, *ζ* is the identification coefficient and has a value between 0 and 1; this coefficient is usually considered 0.5. Also*, ∆*_*min*_ and *∆*_*max*_ are minimum and maximum values of *∆*_*oi*_* (p),* respectively. The values are given in Table 7.

**Table 7:** Grey relation coefficient.

No. of trial	Young’s modulus (MPa)	Strength (MPa)	Deformation	Cylindricity (mm)	Circularity (mm)	GRG	Rank
1	0.557	0.746	0.339	1.000	1.000	0.728	2
2	0.341	0.399	0.796	0.343	0.486	0.473	8
3	0.352	0.383	0.490	0.501	0.750	0.495	7
4	0.388	0.387	0.366	0.594	0.791	0.505	6
5	0.604	0.595	1.000	0.389	0.333	0.584	4
6	0.601	0.894	0.340	0.417	0.488	0.540	5
7	0.333	0.333	0.360	0.333	0.621	0.396	9
8	0.821	0.803	0.333	0.920	0.843	0.744	1
9	1.000	1.000	0.352	0.391	0.592	0.667	3

#### Gray relational grade

In general, the Gray Relational Grade (GRG) is used to evaluate the multi-response properties. On the other hand, GRG is the average sum of the GRC, and Eq. (6) is used to determine it^36^.

6 $${\gamma }_{i}=\frac{1}{n}\sum_{i=1}^{n}\zeta \left(p\right)$$

where *n* is the number of prosses parameters. As a result, a larger GRG implies that the process parameter combination is closer to the ideal. After that, all experimental experiments were ranked based on GRG values from 1 to 9, the highest GRG value representing the Optimum run, and it is considered 1st rank. So, the 8th test, which has the highest GRG value, so the 8th experiment has the best characteristics among the other trials.

### Analysis of GRG data

#### Analysis using ANOVA and S/N ratio

Signal-to-noise (S/N) ratio is used to optimize process parameters and examine each parameter's impact on response. In the S/N ratio, the "signal" indicates the desired effect, while the "noise" indicates the undesirable effect for the responses. Therefore, if the S/N ratio is higher, it indicates the optimal conditions. According to the expected quality characteristics of different responses, there are different types of S/N ratios, including larger-is-better, smaller-is-better, and normal-is-better. Where *η* represents the S/N ratio, *yi* represents the response value of the target experiment in the orthogonal array, *yn* shows the variance, and *n* is the number of experiments

Larger-is-better:

7 $$\eta =-10\,\mathrm{ log}\left[\frac{1}{n}\sum_{i=1}^{n}\frac{1}{{y}_{i}^{2}}\right]$$

Smaller-is-better:

8 $$\eta =-10\,\mathrm{ log}\left[\frac{1}{n}\sum_{i=1}^{n}{y}_{i}^{2}\right]$$

Normal-is-better:

9 $$\eta =-10\,\mathrm{ log}\left[\frac{1}{n}\sum_{i=1}^{n}{\left({y}_{i}-{y}_{n}\right)}^{2}\right]$$

Analysis of variance (ANOVA) and signal-to-noise ratio (S/N) were used to analyze the data obtained from GRG using MINITAB^®^19.0. To examine the effect of each parameter on GRG, the Taguchi technique was utilized. Due to the higher the GRG value, the desired responses improve, so “larger-is-better” was used to maximize the GRG to optimize the process parameters. The S/N ratios and response table of means for GRG are shown in Tables 8 and 9, respectively. These tables show the significance of parameters by utilizing rank, and delta represents the difference between the highest and lowest average. According to the results, it can be said that the print speed and printing temperature have the most significant impact compared to the chamber temperature and layer thickness on GRG.

**Table 8:** Response table for signal to noise ratios of GRG.

Level	Chamber temperature (°C)	Printing temperature (°C)	Layer thickness (mm)	Print speed (mm/min)
1	− 5.123	− 5.579	− 3.559	− 3.649
2	− 5.319	− 4.581	− 5.318	− 6.634
3	− 4.711	− 4.993	− 6.275	− 4.870
Delta	0.609	0.998	2.716	2.985
Rank	4	3	2	1

**Table 9:** Response table for means of GRG.

Level	Chamber temperature (°C)	Printing temperature (°C)	Layer thickness (mm)	Print speed (mm/min)
1	0.5653	0.5430	0.6707	0.6597
2	0.5430	0.6003	0.5483	0.4697
3	0.6023	0.5673	0.4917	0.5813
Delta	0.0593	0.0377	0.2934	0.1900
Rank	3	4	2	1

**Figure 7:**
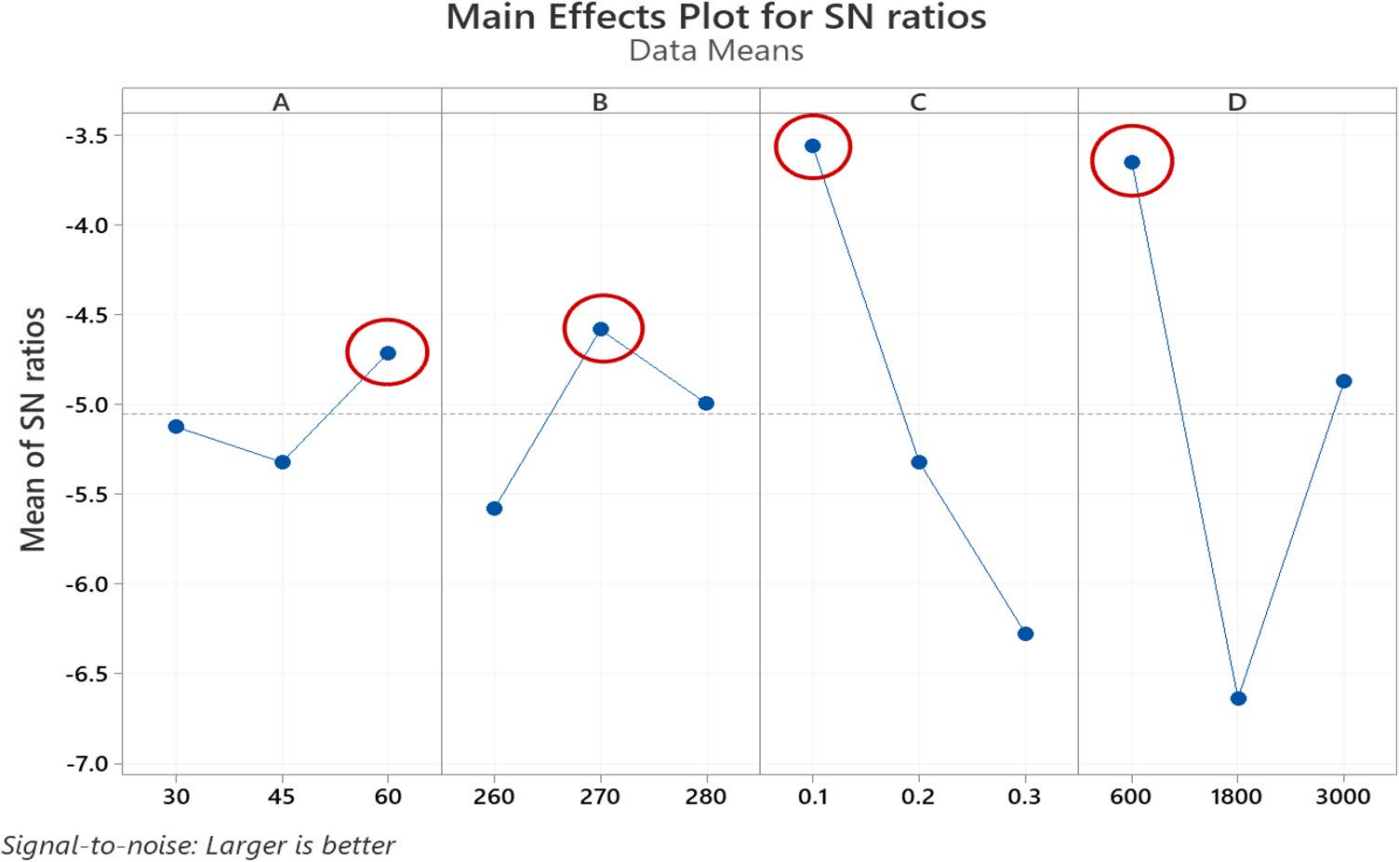
Main Effects Plot for S/N ratios GRG.

**Figure 8:**
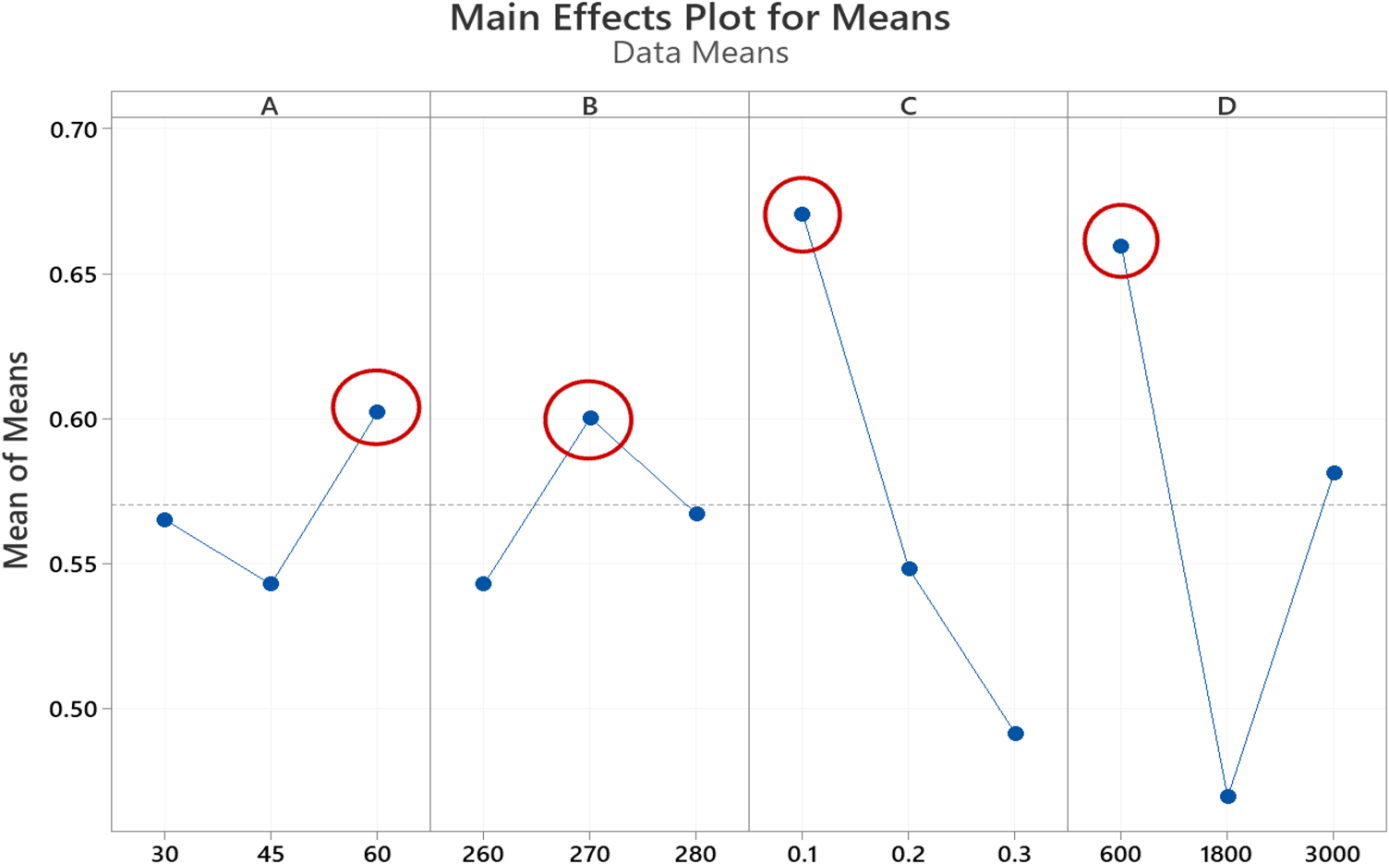
Main Effects Plot for means GRG.

**Figure 9:**
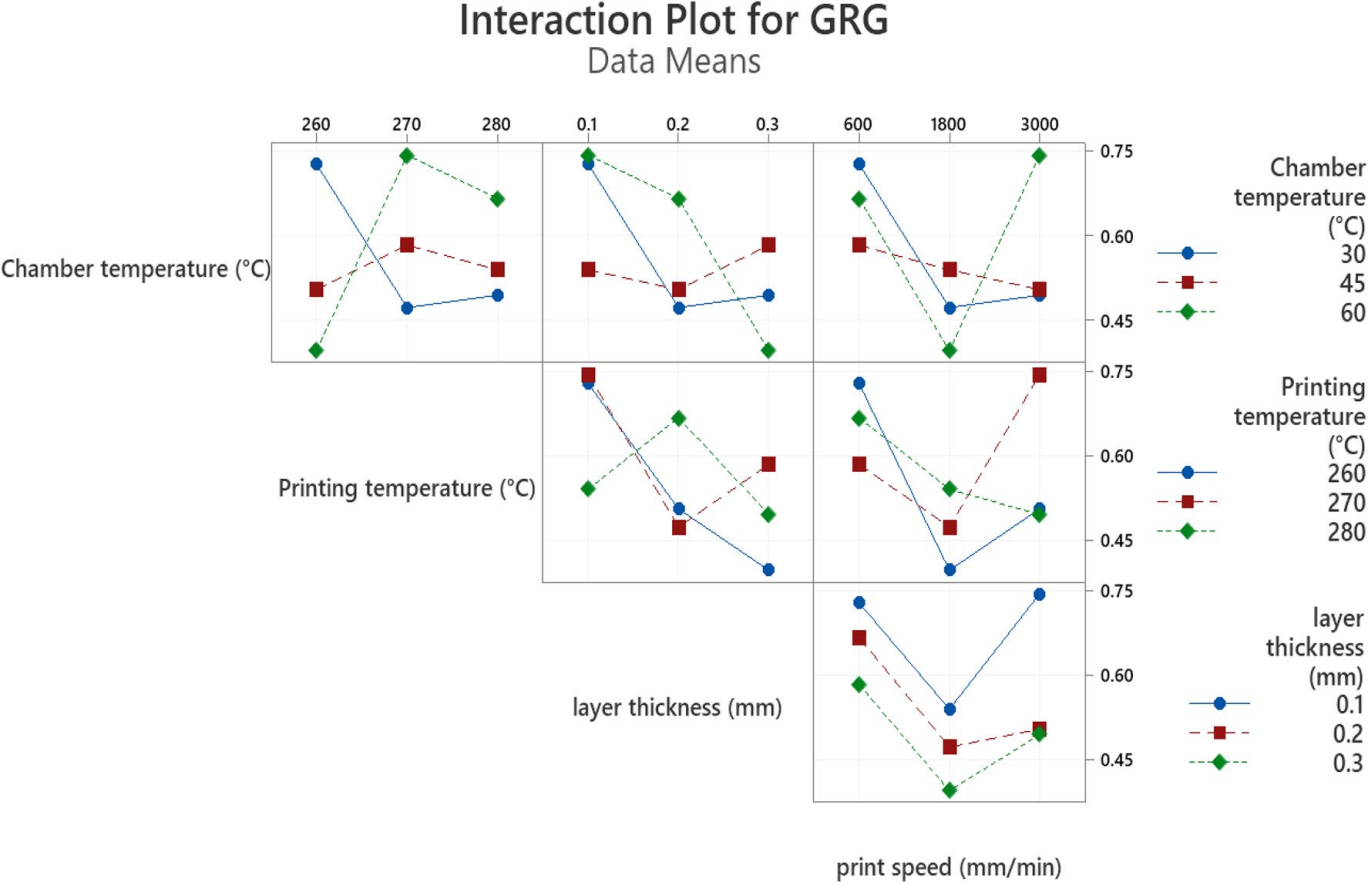
Interaction plots for GRG.

**Figure 10:**
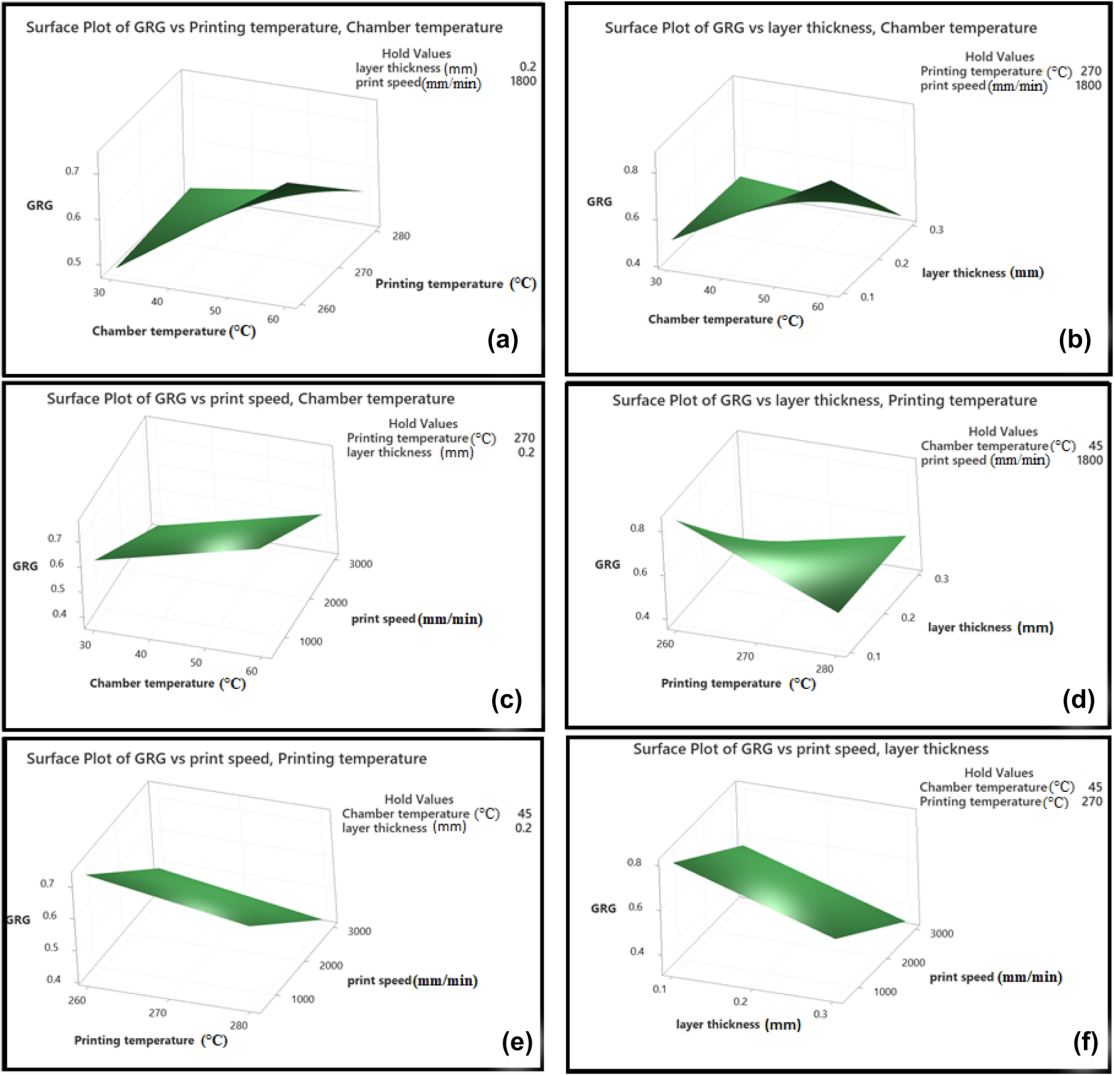
GRG Surface plots. (**a**) chamber temperature and printing temperature, (**b**) chamber temperature and layer thickness, (**c**) chamber temperature and print speed, (**d**) printing temperature and layer thickness, (**e**) printing temperature and print speed, (**f**) layer thickness and print speed, on GRG.

The S/N diagram was used to analyze the data and determine optimal parameters using average S/N ratios for responses. As minimization of the output, parameters are required for geometrical accuracy (cylindricity and circularity), the “smaller-is-better,” and to maximization of the output parameters are required for mechanical properties (Young’s modulus, deformation, and strength), the “larger is better” was selected to maximize mathematical expression for the S/N ratio. The best condition is shown by the highest point in the S/N ratio graphic. A B, C, and D represent the chamber temperature, 3D Printing temperature, layer thickness, and print speed in Figs. 9 and 10, respectively. The various levels for each parameter are represented on the horizontal axis, and the vertical axis is the mean S/N ratio. According to the Main Effects Plot for S/N ratio (Fig. 7) and Main Effects Plot for mean diagrams (Fig. 8), it can be seen that chamber temperature is 60 °C, printing temperature 270 °C, layer thickness 0.1 mm, and print speed 600 mm/min is the optimum combination of processes parameters for achieving the maximum GRG.

The impact of each process parameter on the response variables was determined using the ANOVA approach. The results of ANOVA are shown in Table 10. The adjusted sum of squares (Adj SS) was calculated using Eq. (10).

10 $${s}_{T}=\sum_{i=1}^{n}{\left({\eta }_{i}-{\eta }_{j}\right)}^{2}$$

where *η*_*i*_ represent the mean S/N ratio, *η*_*j*_ is the overall mean S/N ratio, and n shows the total number of experiments. DF stands for the degree of freedom, and Contribution is Percentage of contribution of process parameters, the adjusted mean sum of squares is Adj MS, while the variance of the group means and the probability value is F-Value and P-Value, respectively. By investigating the F-value mentioned in Table 10 and considering that the higher the value, the greater the effect of the related parameter, it was determined that print speed, layer thickness, Chamber temperature, and Printing temperature have the most significant effect on the amount of GRG respectively. Contribution Percentages also confirms these results.

**Table 10:** Response of variable parameters of GRG.

Source	DF	Adj SS	Adj MS	F-Value	P-Value	Contribution (%)
A	2	0.005388	0.002694	1.08	0.480	4.7
B	2	0.004968	0.002484	–	–	–
C	2	0.050218	0.025109	9.32	0.097	43.6
D	2	0.054706	0.02735	10.15	0.09	47.4
Pulled error	2	0.004968	0.002484	–	0.520	4.3
Total	8	0.115280		–	–	100.0

Figure 9, shows the interaction between process parameters and GRG values. In an interaction plot, parallel lines imply no interaction. So according to the figure, in all other diagrams except speed, there are no parallel trend lines and the directions are mixed. As a result of this strong evidence that the linear regression model is useless for GRG predictions, the response surface method's linear-interaction model is used. Using a linear-interaction model of RSM, a multi-objective function for GRG was constructed and nominal terms have been removed from the equation^37,38^.

#### Regression modeling of GRG

Response surface regression examines the correlation between variables, which determines the relationship between GRG and process parameters. Also, the best responses can be achieved by finding the best correlation between factors and the best levels of parameters linear-interaction model of the response surface method is used. The regression model is shown in Eq. (11).

11 $$GRG=3.38+0.1082A-0.01211 B-28.76C-0.000098D-0.00033A*B-0.071A*C+0.1141B*C$$

The chamber temperature, printing temperature, layer thickness, and print speed are represented by A, B, C, and D, respectively. The correlation coefficient, often known as R-squared, is a statistical tool that represents the proportion of variation in a dependent variable and ranges from 0 to 100 percent. MINITAB 19.0^®^ software calculates the R-squared value, and the value of this coefficient is 99.20 percent, which indicates a high correlation.

**Figure 11:**
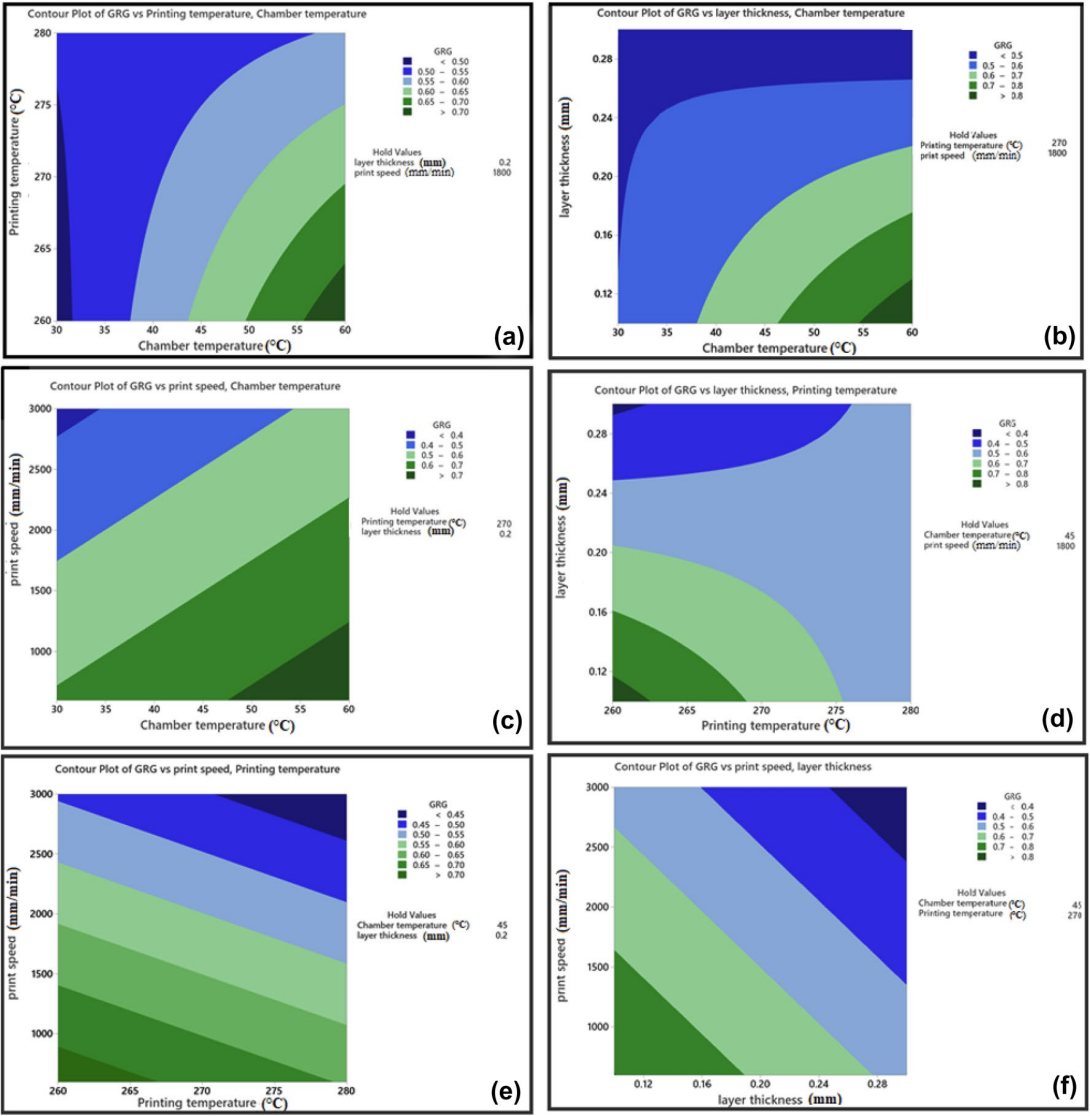
GRG contour plots. The effect of; (**a**) chamber temperature and printing temperature, (**b**) chamber temperature and layer thickness, (**c**) chamber temperature and print speed, (**d**) printing temperature and layer thickness, (**e**) printing temperature and print speed, (**f**) layer thickness and print speed, on GRG.

**Figure 12:**
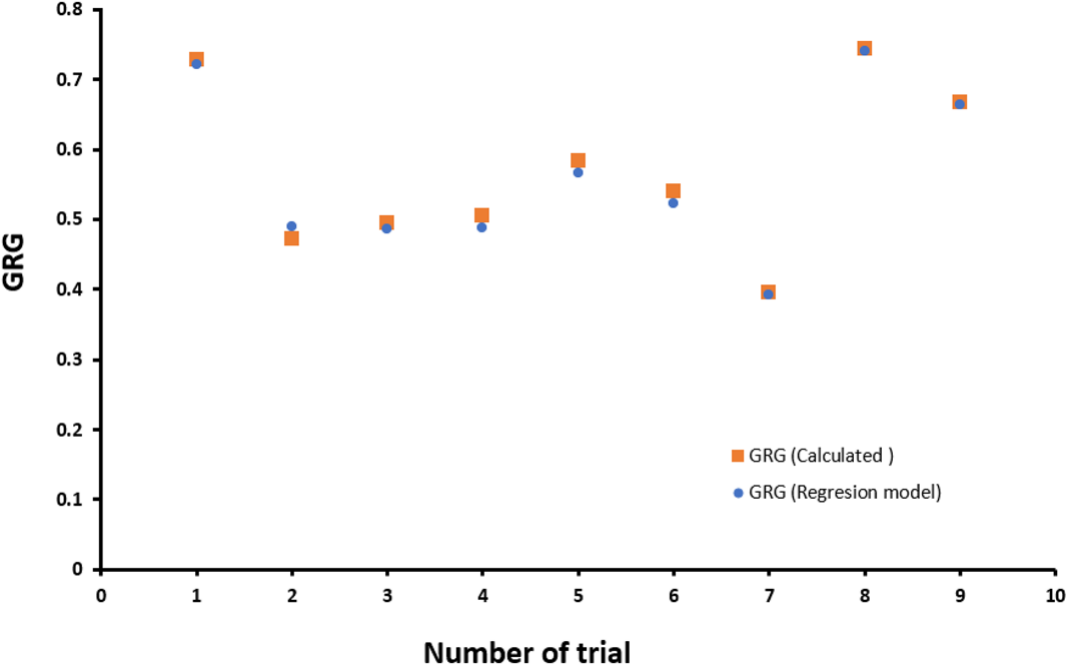
Comparison of GRG values obtained from regression model and obtained from experimental.

Figures 10 and 11 refer to surface diagrams and contour diagrams, respectively, which are graphical images of the regression equation. They show the interactions between two different process parameters on GRG and are made by MINITAB 19.0 software. As can be seen from these graphs, the highest value of GRG is obtained at the lowest values of layer thickness and print speed and the highest values of chamber temperature. Also, as is observed in Fig. 12, by Comparing the GRG values obtained by the experiments and the GRGs calculated by the regression equation, it is determined that the maximum error rate is 3%, indicating that the model is validated.

**Figure 13:**
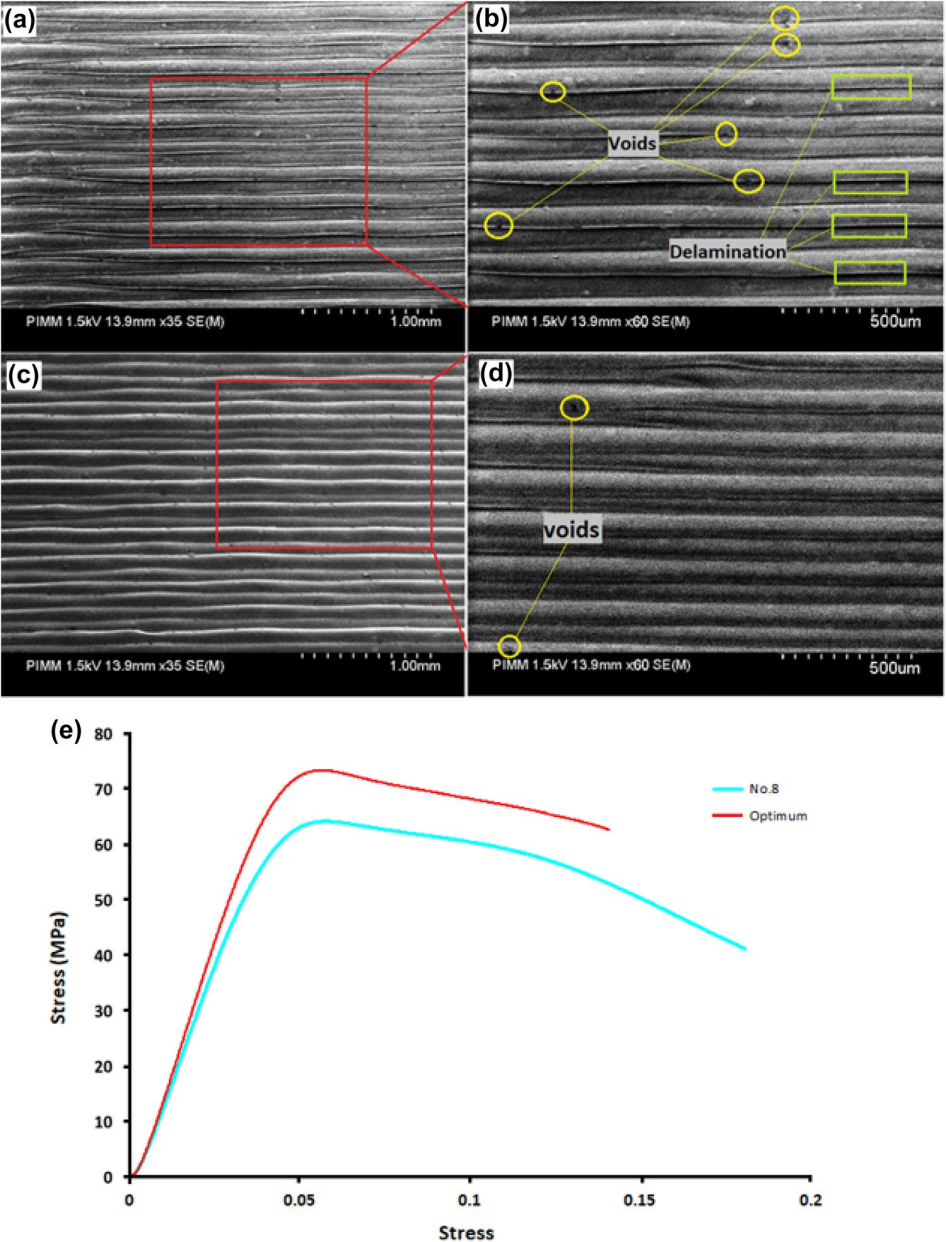
Scanning Electron Microscopy (SEM) images of the samples manufactured by FFF in (**a**,**b**) 8th, (**c**,**d**) and optimum condition, respectively. (**e**) Stress strain curve of initial GRG (8th) and the GRG obtained from the experiment.

In the last step, a confirmation experiment was performed using optimum levels of process parameters (Chamber temperature 60 °C, Printing temperature 270 °C, layer thickness 0.1 mm, and print speed 600 mm/min) to verify this parameter obtained from the GRA and also to evaluate the improvement in responses. To ensure repeatability of the results, five hollow cylindrical parts with optimal parameters were fabricated by the FFF 3D printer. And the predicted Grey relational grade value or *Y*_*predicted*_ is compared to the mean value of the grey relational grade obtained from the confirmation test. Equation (12) is used to calculate the predicted GRG value for optimal parameters.

12 $${Y}_{Predicted}={y}_{m}+\sum_{i=1}^{n}\left({y}_{i}-{y}_{m}\right)$$

13 $$Error \left({\%}\right)=\left[\frac{{GRG}_{Predicted}-{GRG}_{Experimental}}{{GRG}_{Experimental}}\right]*100$$

14 $$Improvment \left(\mathrm{\%}\right)=\left[{GRG}_{Experimental}-{GRG}_{Intitial}\right]*100$$

where *y*_m_ represents the total mean of the GRG, *y*_*i*_ refers to the average GRG at the optimal level, and n is the number of chosen process parameters. Then a compression test was applied to the parts to evaluate the strength, young’s modulus, and elongation of PA6 parts. Also, for measuring geometrical error values such as cylindricity and circularity, 3D printed parts were scanned using Solutionix D500, and the measured value is shown in Table 11. Also, the stress-strain curve of optimum parameters and the 8th trial, which has the most GRG, are compared in Fig. 13e. As it turns out, the mechanical properties such as young’s modulus and strength of the printed part have been improved under optimal conditions. Then, using the values of the obtained responses, the GRG value for the 3D printed piece with optimal parameters was measured using Eqs. (1), (2), (4), (5), and (6). After calculating the experimental GRG, the next step is to calculate the percentage error between the predicted GRG and the experimental GRG. Then the improvement in GRG is evaluated. All the measured values of GRG are shown in Table 12, and by comparing the initial GRG and the GRG obtained from the experiment and using Eq. (13), it was found that the optimum GRG value has improved by 14%. So, the results show that the values of the optimal parameters obtained from the GRA method have improved all the intended responses. Also, by comparing the predicted GRG and the GRG of the experiment (Eq. 14), it was found that the error rate is equal to 5%. Therefore, considering this amount of error, it can be said that there is a good correlation between these values.

**Table 11:** Measured values of responses.

Young’s modulus (MPa)	Strength (MPa)	Deformation	Cylindricity (mm)	Circularity (mm)
1890.22 ± 27.49	73.37 ± 0.19	0.0560 ± 0.00031	0.1528 ± 0.00781	0.10106 ± 0.0111

**Table 12:** Measured values of responses.

	Initial setting	Predicted	Experimental
Chamber temperature (°C)	60		60
Printing temperature (°C)	270		270
Layer thickness (mm)	0.1		0.1
Print speed (mm/min)	3000		600
GRG	0.744	0.823	0.868

Figure 13a–d shows the SEM observation of the samples manufactured in 8th and optimum conditions. More voids and delamination can be seen in the 8th (initial condition) sample compared to the optimum condition 3D printed part. Given the difference in printing speed for fabrication of these parts, the difference in print quality can be attributed to this parameter. One of the critical parameters affected by changing the printing speed is thermal gradient^39^. So, the effect of thermal gradient on delamination between the layers has been emphasized. By increasing the nozzle speed cooling rate will be increased^40^. So optimum part which printed at a lower speed will be under a lower thermal gradient than the 8th. So, more delamination in 8th is justified. Furthermore, the weld interface between filaments plays a vital role in final mechanical properties^41^. As shown in the Fig. 13 weld interface in this figure, it has not been done well considering the presence of some delamination. Few mechanical properties were expected by reducing the weld interface between the two filaments. The compression test results show a decrease in the compressive strength of 8th printed part compared to the optimum part. Furthermore, the geometric accuracy is less due to more delamination in the 8th printed part compared to the condition 3D printed part^42^.

The results of this research enable the designer to produce high-quality parts. In the following, the results of this research and the optimal selection parameters, and its comparison with other articles expressed in the literature section are discussed. It was found that at 270 °C, the amount of GRG was optimum. The optimum 3D printing temperature depends on the type of material used, etc., but at low temperatures, the printed layer is almost solid. The decreased binding force between the layers will be reduced if the new layer is deposited. The adhesion will be very poor, so it causes lower mechanical properties and dimensional accuracy. On the other hand, if the temperature is high, the fluidity is too high, and due to the gravity force, the stability of the geometry decreases. It was found that the 60 °C chamber temperature, which was the highest value considered in this study, is the optimal GRG level. This is because the high chamber temperature is not too high to affect geometric stability. In the mentioned research in the literature, it was observed that the highest temperature followed the best dimensional accuracy in PLA parts with increasing temperature of 3D printing. Another study on PEEK components had similar results to the current work. This can be due to different materials used and different temperature ranges^16,17^. Also, in a study, the effect of chamber temperature on the strength of PEAK components was investigated. Similar results were observed^19^. According to the optimization results, it was found that the amount of GRG at low speeds is higher. Because at high speeds, the printed layers do not have enough time to solidify. The following layers are deposited on the previous layers, and the piece becomes deformed within a short time interval. Other PEEK and PC/ABS blend parts research indicated that higher printing speed values enhanced and optimized strain, strength, and stiffness. The difference in results might be due to limitations in the cylinder design, the type of material utilized, and the responses selected^17,19^. The effect of layer thickness as one of the most critical parameters in improving parts’ mechanical properties and accuracy was investigated. It was found that the amount of GRG is higher at lower layer thickness. A thicker layer thickness results in higher temperature gradients between the layers, which leads to more deformation. Also, as the number of deposited layers increases, more interfaces appear, and adhesion reduces. However, as indicated in the literature, different results and similar results were observed in articles with different materials. And the reason can be the difference in the desired responses and the difference in the selected material. Another reason could be other selected parameters because all parameters affect each other^15,16,19,21^.

## Conclusions

The present paper uses multi-response optimization utilizing the GRA method to analyze the variation of four input parameters, including chamber temperature, printing temperature, layer thickness, and print speed, to achieve the best mechanical properties and geometrical accuracy in the FFF process cylindrical parts made of PA6. The mechanical properties and geometrical accuracy are characterized through cylindricity, circularity, strength, Young’s modulus, and deformation. These five output parameters represent the expected responses. For this purpose, it was to find the optimum values of the processing parameters to improve all the responses simultaneously. It was determined that the highest GRG belongs to the 8th experiment. Then, to find the optimal parameters, GRG data were analyzed by ANOVA and S/N analysis, and it was determined that the optimal conditions for improving GRG would be obtained at Chamber temperature of 60 °C, Printing temperature of 270 °C, the layer thickness of 0.1 mm and print speed of 600 mm/min. Finally, a verification test was performed according to the optimal parameters, and new components were examined. Finally, by comparing the initial GRG and the GRG obtained from the experiments, it was observed that the GRG value had improved 14%. Also, by comparing the predicted GRG and the GRG of the experiment, it was found that the error rate is equal to 5%. Therefore, considering this amount of error, it is proved that there is a good correlation between these values.

Finally, the results were discussed, and it is clear that:

1. The optimum GRG level was discovered at a chamber temperature of 60 °C, which may be because the temperature in the chamber is not too high to impact geometric stability.
2. The amount of GRG will be optimum at 270 °C printing temperature. However, the optimum 3D printing temperature varies depending on the material used, etc. At low temperatures, the printed layer is almost solid, and if a new layer is deposited, the binding force between the layers will be reduced. Adhesion will be poor, resulting in lower mechanical properties and dimensional accuracy. On the other side, if the temperature is too high, the fluidity is too great, and the geometry’s stability is reduced owing to gravity.
3. According to the optimization results, the quantity of GRG was determined to be greater at low speeds. This can be explained by the fact that the printed layers do not have enough time to solidify at high speeds. The following layers are deposited on top of the previous layers, causing the piece to deform.
4. The influence of layer thickness was examined, and it was discovered that the amount of GRG is more significant at lower layer thickness. As the number of deposited layers increases, more surfaces appear, and adhesion will be decrease, a wider layer thickness resulting in higher temperature gradients between the layers, which leads to increased deformation.
